# London Homœopathic Hospital

**Published:** 1903-07-04

**Authors:** 


### LONDON HOMŒOPATHIC HOSPITAL.

### TRIENNIAL FESTIVAL DINNER.

Earl Cawdor, Vice-President and Treasurer, took the chair at the triennial festival dinner of the London Homœopathic Hospital, at the Hotel Cecil, on June 25th.

After the loyal toasts the chairman gave "Prosperity to the Hospital." The toast itself, he said, was a plea to those who were invited to drink it to make that prosperity possible. The hospital was now 53 years old. There were 123 hospitals in London, and the Homœopathic was older than 84 of them. Looking back a year or two, they had in 1898 1,100 in-patients and 32,000 out-patients. In 1901 the number of in-patients remained the same because that was the extent of their capacity, but the 32,000 out-patients had grown to 40,000. In 1902 they had a "spring cleaning" and the number of out-patients was reduced to 38,000. During the year there were 480 surgical operations, of which 230 were major operations. This was a tale of usefulness among the poorer classes of which they were proud. But their plea was not only as a charitable institution. The hospital was that, and he hoped it always would be, but it was also the centre of homœopathy in England, and they could justify their case as doing good work. He hoped that among the many claims brought to their notice, homœopathists would not neglect that Turning to the other side of the account, he was sorry to say they had a debt of £12,000. Of that £3,000 was an overdraft at the bank, and he hoped that at least they would wipe that out and something more. £9,085 was drawn from their reserve funds as authorised by the annual meeting some years ago. If they cast their eyes back over the accounts from 1896 they would see that there had been an annual average deficit of £2,700. Nations could not get on without a national debt and it was said that hospitals could not exist without a debt either, but he should like to try. (Laughter and cheers.) Their increase of expenditure and usefulness had brought with it increase of work. They had still many good friends though they missed many old faces. Among the most generous of their friends was Lord Dysart, and to meet the deficit he had promised them £2,000 if they got £10,000 before next Christmas or £1,000 if they got £5,000. (Cheers.) Their heartiest thanks were due to Lord Dysart, not only for his help but for his encouragement to others. Now they must look round for new friends to help them. Already this year they had a good deal of a satisfactory character on which to congratulate themselves. The Duke of Teck had become a patron. The late Duchess of Teck—Princess Mary—had always been one of their warmest supporters—(cheers)—and they liked to watch heredity going in the right course. (Hear, hear.) Lord Napier of Magdala had become a Vice-President, as had also Sir Edward Thornton; Earl Morley, the Chairman of Committees in the House of Lords, and Lord Windsor, the Chairman of the Board of Works—so they were not slipping into the background. (Hear, hear.) He must say one word about the Ladies' Guild. (Cheers.) It now numbered 300, and was finding them £150 a year in subscriptions. The Hampstead branch maintained a bed at a cost of £50, and the Highgate branch a cot at £35. So there was no doubt they had the ladies behind them, and he tendered them their warmest thanks. Summing up he might say they were doing much good work; they had much help from many good friends, and he was glad to say most from those who knew the hospital best—not by outsiders. This was the most hopeful sign of all. He tendered to their excellent medical staff their most grateful thanks not only for their medical work, but for the way in which they buckled to and got financial help for the hospital, with very satisfactory results. The institution was doing good work, and wanted to do more. Their plea for help was not for a moribund, a tottering or a weak concern, but for one full of life and vigour. (Cheers.)

Sir Henry Tyler proposed the toast of "Homœopathic Hospitals and Dispensaries throughout the Empire," and this was responded to by Councillor G. G. Crespin, treasurer of the Homœopathic Hospital, Melbourne, and by Dr. Deck, of Sydney.

The Secretary Superintendent (Mr. G. A. Cross) then read the list of subscriptions, which totalled £5,297, plus a further £1,000 by Earl Dysart on condition that the amount was made up to £10,000 by Christmas.

Other toasts followed.

